# Breast‐conserving therapy shows better prognosis in mucinous breast carcinoma compared with mastectomy: A SEER population‐based study

**DOI:** 10.1002/cam4.3202

**Published:** 2020-06-08

**Authors:** Ping Yu, Peng Liu, Yutian Zou, Xinhua Xie, Hailin Tang, Na Li, Xiaoming Xie, Feng Ye

**Affiliations:** ^1^ Department of Breast Oncology Sun Yat‐Sen University Cancer Center State Key Laboratory of Oncology in South China Collaborative Innovation Center for Cancer Medicine Guangzhou China

**Keywords:** breast‐conserving therapy, mucinous breast carcinoma, prognosis, SEER

## Abstract

**Background:**

Mucinous breast carcinoma (MBC) is a relatively rare pathological type of breast cancer. Compared with mastectomy in MBC, the effect and safety of breast‐conserving therapy (BCT) remains unclear. Therefore, we investigated the long‐term prognosis of BCT and mastectomy in T1‐2 stage mucinous breast carcinoma via the Surveillance, Epidemiology, and End Results (SEER) database.

**Methods:**

Totally, 8830 patients who were diagnosed of mucinous breast carcinoma between 2004 and 2014 from SEER database were reviewed retrospectively. Cox proportional hazards model and Kaplan‐Meier method were performed for evaluating the relationship between surgical method and prognosis.

**Results:**

One thousand three hundred and twenty (14.9%) patients underwent mastectomy and 7510 (85.1%) underwent BCT. The median follow‐up time was 77 months. There were more non‐Hispanic white, married, and younger (<65 years) patients, as well as lower stage of tumor sizes, lymph nodes and more favorable histologic grade, ER positive, and PR positive in BCT group (*P* < .05). Patients in BCT group had relatively better overall survival (OS) than those in mastectomy group. The risk of death from any cause in BCT group was lower than that in mastectomy group significantly (HR = 0.786, 95% CI: 0.703‐0.879, *P* < .001), while no difference significantly was observed in breast cancer‐specific survival (BCSS) between BCT and mastectomy groups. In stratified analysis according to T stage, BCT group had better OS than mastectomy group for patients of T1 stage (HR = 0.679, 95% CI: 0.589‐0.781, *P* < .001) or T2 stage (HR = 0.769, 95% CI: 0.646‐0.915, *P* = .003). In stratified analysis according to the different ages, BCT showed OS benefit in patients at the age of 50‐64 years (HR = 0.587, 95% CI: 0.408‐0.846, *P* = .004) and the age of 65‐79 years (HR = 0.636, 95% CI: 0.535‐0.758, *P* = .001). For patients younger than 50 years or not younger than 80 years, there was no difference significantly observed in OS between BCT and mastectomy groups (*P* > .05).While for patients who received BCT, the use of radiotherapy showed OS benefit.

**Conclusions:**

This large population‐based study indicated patients who received BCT had better prognosis than those received mastectomy in T1‐2 stage MBC, especially in patients at the age of 50‐79 years. The use of radiotherapy showed OS benefit in patients receiving BCT. Breast‐conserving therapy might be preferred over mastectomy especially in locoregional treatment of T1‐2 stage MBC.

## Graphical abstract

This large population‐based study confirmed that patients with BCT had better prognosis than those with mastectomy in T1‐2 stage MBC, especially in patients with age of 50‐79 years. The use of radiotherapy show OS benefit in patients receiving BCT. Breast conserving therapy should be preferred over mastectomy in locoregional treatment of T1‐2 stage MBC.

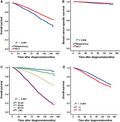

## 1 INTRODUCTION

Mucinous breast carcinoma (MBC), termed as colloid cancer, is a relatively rare pathological type of breast cancer. Its incidence accounts for approximately 1% to 7% of the primary breast cancers.^1^, ^2^, ^3^, ^4^, ^5^, ^6^

Compared with other common types of breast cancer such as infiltrating ductal cancer (IDC), MBC has some different clinical features. MBC usually occurs in postmenopausal and elderly patients and generally has a favorable prognosis. MBC has a higher proportion of estrogen receptor (ER) and progesterone receptor (PR) positivity, better differentiation and a lower rate of lymph node metastases.^1^, ^2^, ^7^, ^8^, ^9^

On account of its rarity, most studies about MBC have either small samples or a relatively short‐term follow‐up. So the evidence of clinical factors and prognosis of MBC is weak. At present, the guidelines recommendation of locoregional and systemic adjuvant treatment of MBC are based on the data of IDC. Standard IDC surgery is also suitable for MBC patients. Mastectomy and breast‐conserving therapy (BCT) plus radiation therapy could achieve similar prognosis in women diagnosed with early stage breast cancer in some randomized controlled trials.^10^, ^11^Some researchers have recommended that MBC should be treated less aggressively such as BCT than IDC due to more favorable clinicopathologic features of MBC.^8^, ^12^, ^13^ However, the prognosis of BCT and mastectomy in MBC remains unclear. The use of BCT in MBC has been questioned.

Therefore, the objective of this large population‐based study was to investigate the long‐term prognosis of BCT and mastectomy in T1‐2 stage mucinous breast carcinoma using the Surveillance, Epidemiology, and End Results (SEER) database.

## 2 METHODS

### 2.1 Patient selection

The SEER custom database (with additional treatment fields) (http://www.seer.cance r.gov) we used was released in November 2018, which contained the data from 18 population‐based cancer registries.

Patients who were diagnosed of mucinous breast carcinoma between 2004 and 2014 were eligible. Other inclusion criteria involved as follows (1) female; (2) were older than 18 years; (3) T1‐2 breast cancer; (4) breast cancer as a primary cancer diagnosis; (5) underwent mastectomy or breast‐conserving surgery. The exclusion criteria involved as follows: (1) underwent prophylactic mastectomy; (2) synchronic bilateral breast cancers (3) history of malignant tumors other than breast cancer; (4) confirmed metastasis or recurrence at the first diagnosis; (5) complete data cannot be achieved. This research was approved by the Research Ethics Committee in Sun Yat‐sen University Cancer Center.

### 2.2 Data collection and outcomes

The following factors were extracted: demographic factors (year of diagnosis, age at diagnosis, race, and marital status), clinicopathological factors (tumor size (T stage), lymph node status (N stage), TNM stage, histologic grade, estrogen receptor (ER), progesterone receptor (PR)), therapeutic factors (surgery of primary site in terms of the “breast surgery codes C50.0‐C50.9”, radiotherapy, chemotherapy), and survival factors (death events and survival months). According to the 6th edition of the American Joint Committee on Cancer staging system, the patients’ pathological TNM stages were confirmed.

The primary end point was overall survival (OS) which was calculated from the date of diagnosis to the date of death from any cause or last follow‐up. And the secondary end point was breast cancer‐specific survival (BCSS) which was calculated from the date of diagnosis to the date of death due to breast cancer.

### 2.3 Statistical analysis

In descriptive statistics, the continuous variables were described as median and range. The categorical ones were described as frequencies and percentages. Chi‐square test was applied to compare categorical data. Kaplan‐Meier method and log‐rank test were used to get the OS curves and compare the differences. Univariate and multivariate Cox model were performed to assess the risk factors for OS and BCSS. The statistical analyses were done by the SPSS, version 22.0 (SPSS Inc).A two‐side *P* < .05 was thought to be significant statistically.

## 3 RESULTS

### 3.1 Baseline characteristics

In total, 8830 patients who were diagnosed of mucinous breast carcinoma between 2004 and 2014 were included for analysis. The process of patient selection was presented in supplementary Figure 1. Table 1 summarized the clinicopathological characteristics according to the surgical methods. Among 8830 patients, 1320 (14.9%) underwent mastectomy, 7510 (85.1%) underwent BCT. Patients underwent BCT more frequently than mastectomy. The median age was 67 years (range, 23‐103 years). Compared with patients in mastectomy group, there were younger (<65 years), more non‐Hispanic white, married patients as well as lower stage of tumor sizes, lymph nodes and more favorable histologic grade, ER positive, PR positive in BCT group (*P* < .05). Patients in BCT group were more likely to receive radiotherapy (*P* < .001). While patients in mastectomy group were more likely to receive chemotherapy (*P* < .001).

**FIGURE 1:**
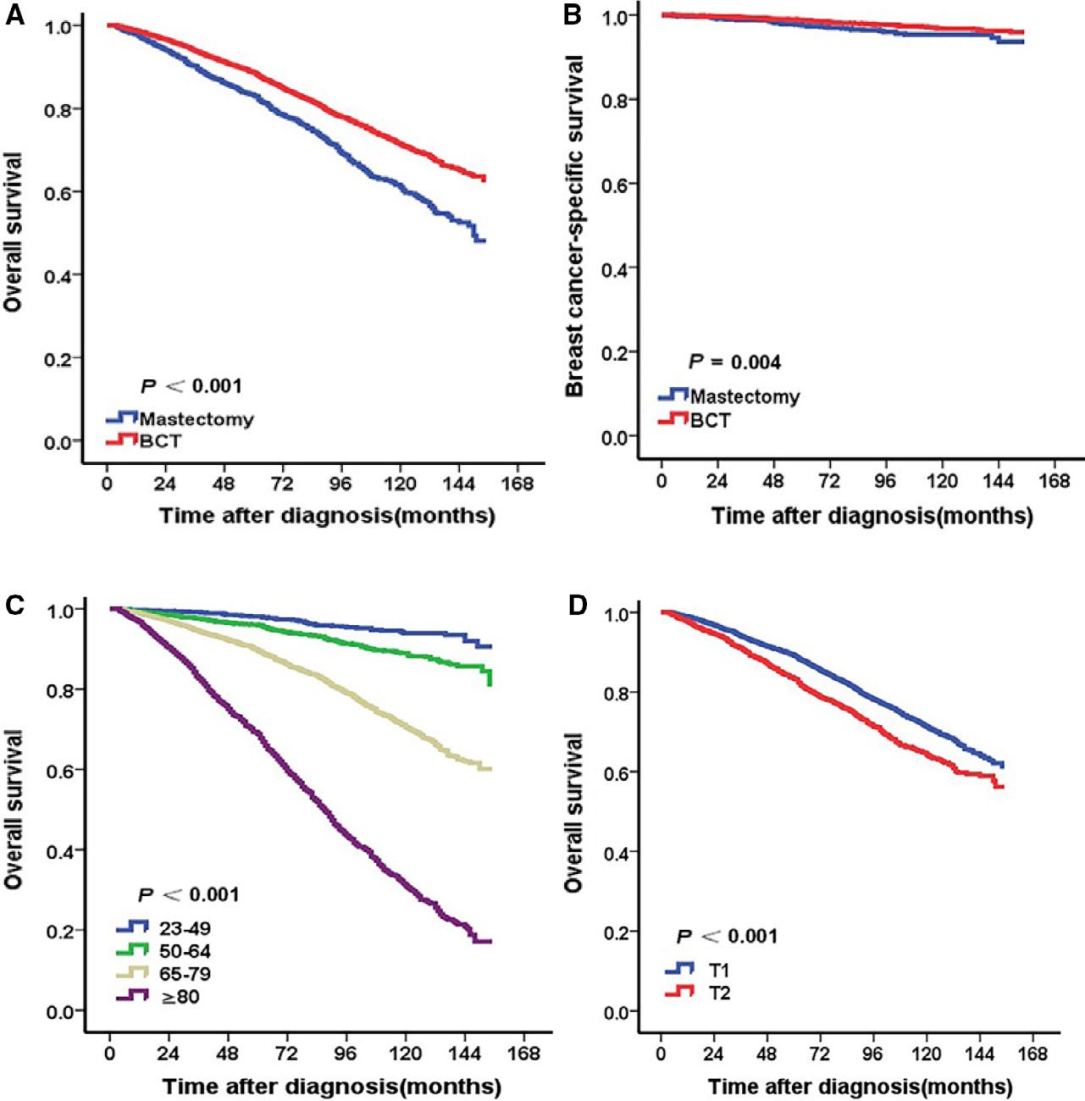
Kaplan‐Meier curves of survival for patients according to surgical methods, age, and T stage. BCT group had better OS (A) and BCSS (B) than mastectomy group. The patients with age of 23‐49 years (C), T1 stage (D) had better OS. (all *P* < .05

**TABLE 1:** Baseline characteristics of mucinous breast carcinoma patients

Characteristic	Patients, n (%)			*P* ^a^
	All	BCT	Mastectomy	
No. of patients	8830 (100)	7510 (85.1)	1320 (14.9)	
Year of diagnosis				<0.001
2004‐2006	2754 (31.2)	2187 (29.1)	567 (43.0)	
2007‐2009	2403 (22.7)	1990 (26.5)	413 (31.3)	
2010‐2014	3673 (41.6)	3333 (44.4)	340 (25.8)	
Age (years)				<0.001
18‐49	1045 (11.8)	859 (11.4)	186 (14.1)	
50‐64	2211 (25.0)	1942 (25.9)	269 (20.4)	
65‐79	3693 (41.8)	3134 (41.7)	559 (42.3)	
≥80	1881 (21.3)	1575 (21.0)	306 (23.2)	
Race				<0.001
NHW	6354 (72.0)	5482 (73.0)	872 (66.1)	
NHB	855 (9.7)	691 (9.2)	164 (12.4)	
NHAIAN	37 (0.4)	30 (0.4)	7 (0.5)	
NHAPI	780 (8.8)	643 (8.6)	137 (10.4)	
Hispanic	775 (8.8)	638 (8.5)	137 (10.4)	
Unknown	29 (0.3)	26 (0.3)	3 (0.2)	
Marital status				0.001
Married	4189 (47.4)	3615 (48.1)	574 (47.4)	
Single	1103 (12.5)	910 (12.1)	193 (14.6)	
Widowed	2225 (25.2)	1850 (24.6)	375 (28.4)	
Divorced	847 (9.6)	737 (9.8)	110 (8.3)	
Unknown	466 (5.3)	398 (5.3)	68 (5.2)	
Histologic grade				<0.001
G1	4963 (56.2)	4334 (57.7)	629 (47.7)	
G2	2558 (29.0)	2143 (28.5)	415 (31.4)	
G3	273 (3.1)	206 (2.7)	67 (5.1)	
Unknown	1036 (11.7)	827 (11.0)	209 (15.8)	
T stage				<0.001
T1	6659 (75.4)	5914 (78.7)	745 (56.4)	
T2	2171 (24.6)	1596 (21.3)	575 (43.6)	
N stage				<0.001
N0	8253 (93.5)	7174 (95.5)	1079 (81.7)	
N1	494 (5.6)	308 (4.1)	186 (14.1)	
N2	62 (0.7)	23 (0.3)	39 (3.0)	
N3	21 (0.2)	5 (0.1)	16 (1.2)	
Stages				<0.001
I	6411 (72.6)	5745 (76.5)	666 (50.5)	
II	2336 (26.5)	1737 (23.1)	599 (45.4)	
III	83 (0.9)	28 (0.4)	55 (4.2)	
ER				<0.001
Negative	146 (1.7)	110 (1.5)	36 (2.7)	
Positive	8297 (94.0)	7134 (95.0)	1163 (88.1)	
Unknown	387 (4.4)	266 (3.5)	121 (9.2)	
PR				<0.001
Negative	868 (9.8)	705 (9.4)	163 (12.3)	
Positive	7505 (85.0)	6481 (86.3)	1026 (77.7)	
Unknown	455 (5.2)	324 (4.3)	131 (9.9)	
Chemotherapy				<0.001
No	7887 (89.3)	6792 (90.4)	1095 (83.0)	
Yes	943 (10.7)	718 (9.6)	225 (17.0)	
Radiotherapy				<0.001
No	3656 (41.4)	2742 (32.9)	1184 (41.4)	
Yes	5174 (58.6)	5038 (67.1)	5174 (58.6)	

Abbreviations: BCT, Breast‐conserving therapy; HER‐2, Human epidermal growth factor receptor‐2; HR, Hormone receptor.

^a^ Using Chi‐squared test or Fish exact test. *P* < .05 was considered statistically significant. Compared with patients in mastectomy group, there were younger (<65 years), more non‐Hispanic white, married patients as well as lower stage of tumor sizes, lymph nodes and more favorable histologic grade, ER positive, PR positive in BCT group. Patients in BCT group were more likely to receive radiotherapy. While patients in mastectomy group were more likely to receive chemotherapy.

### 3.2 Univariate and multivariate analysis of OS and BCSS

The median follow‐up time was 77 months (interquartile range 45‐112 months). The 5‐year and 10‐year OS rates in BCT and mastectomy groups were 88.6% vs 83.1% and 71.3% vs 61.1% respectively. The 5‐year and 10‐year BCSS rates in BCT and mastectomy groups were 98.8% vs 97.5% and 96.7% vs 94.6%.

Patients in the BCT group at a younger age or T1 stage had relatively better OS than those in mastectomy group at an older age or T2 stage (all *P* < .05) (Figure 1A,C,D). Adjusting for the significant prognostic variables (race, age, marital status, T stage, N stage, ER, PR, surgery, chemotherapy, and radiotherapy) in univariate analysis, multivariate analysis indicated that the mortality risk from any cause in BCT group was lower than that in mastectomy group significantly (HR = 0.754, 95% CI: 0.697‐0.889, *P* < .001). Besides, patients at a younger age, T1 stage, lower N stage, positive ER status and radiotherapy had better OS relatively (all *P* < .05). The patients with chemotherapy did not achieve OS benefit than those without chemotherapy (HR = 0.860, 95%CI:0.684‐1.080, *P* = .194) (Table 2). There was no difference significantly in OS among different histologic grade or PR status (*P* > .05) (Table 2). Non‐Hispanic Asian or Pacific Islander and married patients had better OS than other groups (all *P* < .05) (Table 2). Adjusting for the significant prognostic variables (age, race, marital status, histologic grade, T stage, N stage, ER, PR, chemotherapy, and radiotherapy), multivariate analysis indicated no difference significantly in BCSS between BCT and mastectomy groups (HR = 1.232, 95% CI: 0.843‐1.800, *P* = .282) (Table 2), although Kaplan‐Meier curve showed patients in BCT group had relatively better BCSS than those in mastectomy group (*P* < .05) (Figure 1B).

**TABLE 2:** Univariate and multivariate analyses of prognostic factors for overall survival, breast cancer‐specific survival

Variable	OS				BCSS			
	Univariate		Multivariate^a^		Univariate		Multivariate^b^	
	HR (95%CI)	*P* ^†^	HR (95%CI)	*P* ^†^	HR (95%CI)	*P* ^†^	HR (95%CI)	*P* ^†^
Year of diagnosis								
2004‐2006	Ref.	Ref.	—	—	Ref.	Ref.	—	—
2007‐2009	1.080 (0.970‐1.203)	0.159	—	—	1.194 (0.849‐1.680)	0.307	—	—
2010‐2014	1.031 (0.903‐1.177)	0.651	—	—	1.054 (0.698‐1.591)	0.802	—	—
Age								
23‐49	Ref.	Ref.	Ref.		Ref.	Ref.	Ref.	
50‐64	2.036 (1.476‐2.808)	<0.001	2.029 (1.464‐2.812)	<0.001	0.763 (0.437‐1.332)	0.341	0.968 (0.546‐1.714)	0.910
65‐79	5.644 (4.203‐7.580)	<0.001	4.960 (3.645‐6.750)	<0.001	1.119 (0.686‐1.827)	0.652	1.707 (0.984‐2.960)	0.057
≥80	19.170 (14.296‐25.707)	<0.001	13.186 (9.626‐18.063)	<0.001	2.873 (1.757‐4.697)	<0.001	3.821 (2.096‐6.964)	<0.001
Race								
NHW	Ref.	Ref.	Ref.		Ref.	Ref.	Ref.	
NHB	0.987 (0.847‐1.149)	0.863	1.045 (0.896‐1.219)	0.574	1.491 (0.981‐2.266)	0.061	1.354 (0.883‐2.076)	0.164
NHAIAN	1.317 (0.728‐2.384)	0.362	1.814 (0.998‐3.297)	0.051	1.299 (0.182‐9.286)	0.794	1.302 (0.179‐9.496)	0.795
NHAPI	0.360 (0.284‐0.456)	<0.001	0.582 (0.458‐0.739)	<0.001	0.440 (0.216‐0.897)	0.024	0.460 (0.222‐0.954)	0.037
Hispanic	0.736 (0.615‐0.882)	0.001	0.924 (0.770‐1.109)	0.396	0.750 (0.416‐1.353)	0.339	0.726 (0.398‐1.322)	0.295
Unknown	0.015 (0.001‐3.689)	0.806	0.001 (0.001‐1.939)	0.842	0.022 (0.005‐7.842)	0.939	0.002 (0.001‐1.459)	0.940
Marital status								
Married	Ref.	Ref.	Ref.		Ref.	Ref.	Ref.	
Single	1.178 (0.996‐1.393)	0.055	1.291 (1.088‐1.531)	0.003	1.716 (1.120‐2.630)	0.013	1.509 (0.971‐2.344)	0.048
Widowed	3.167 (2.856‐3.511)	<0.001	1.446 (1.293‐1.616)	<0.001	1.795 (1.271‐2.536)	0.001	1.038 (0.029‐1.518)	0.849
Divorced	1.424 (1.200‐1.690)	<0.001	1.304 (1.098‐1.550)	0.003	1.170 (0.680‐2.012)	0.571	1.110 (0.641‐1.920)	0.710
Unknown	1.759 (1.421‐2.177)	<0.001	1.287 (1.038‐1.596)	0.022	1.813 (0.983‐3.344)	0.057	1.425 (0.766‐2.650)	0.263
Histologic grade								
G1	Ref.	Ref.	—	—	Ref.	Ref.	Ref.	
G2	1.018 (0.918‐1.129)	0.736	—	—	1.422 (1.024‐1.976)	0.036	1.191 (0.851‐1.665)	0.308
G3	1.050 (0.813‐1.357)	0.709	—	—	2.974 (1.690‐5.236)	<0.001	1.967 (1.078‐3.590)	0.028
Unknown	1.049 (0.919‐1.198)	0.475	—	—	1.339 (0.873‐2.054)	0.81	1.221 (0.792‐1.881)	0.366
T stage								
T1	Ref.	Ref.	Ref.		Ref.	Ref.	Ref.	
T2	1.362 (1.235‐1.503)	<0.001	1.272 (1.148‐1.411)	<0.001	2.643 (1.982‐3.524)	<0.001	2.072 (1.518‐2.829)	<0.001
N stage								
N0	Ref.	Ref.	Ref.		Ref.	Ref.	Ref.	
N1	1.081 (0.899‐1.300)	0.047	1.314 (1.018‐1.596)	0.006	2.253 (1.442‐3.519)	<0.001	1.582 (0.967‐2.586)	0.068
N2	1.138 (0.685‐1.892)	0.617	1.891 (1.117‐3.199)	0.018	6.087 (2.855‐12.980)	<0.001	4.312 (1.881‐9.885)	0.001
N3	0.949 (0.356‐2.531)	0.916	1.270 (0.470‐3.429)	0.637	5.455 (1.352‐22.005)	0.017	2.932 (0.677‐12.702)	0.150
Surgery								
Mastectomy	Ref.	Ref.	Ref.		Ref.	Ref.	Ref.	
BCT	0.666 (0.598‐0.740)	<0.001	0.754 (0.697‐0.889)	<0.001	0.614 (0.439‐0.858)	0.004	1.232 (0.843‐1.800)	0.282
ER								
Negative	Ref.	Ref.	Ref.		Ref.	Ref.	Ref.	
Positive	0.906 (0.652‐1.261)	0.560	0.654 (0.457‐0.936)	0.020	0.311 (0.164‐0.589)	<0.001	0.457 (0.213‐0.982)	0.045
Unknown	1.459 (1.014‐2.099)	0.042	0.865 (0.466‐1.605)	0.645	0.387 (0.164‐0.910)	0.030	0.819 (0.092‐7.328)	0.858
PR								
Negative	Ref.	Ref.	Ref.		Ref.	Ref.	Ref.	
Positive	0.963 (0.833‐1.112)	0.605	1.016 (0.869‐1.188)	0.843	0.597 (0.405‐0.881)	0.009	0.816 (0.518‐1.283)	0.378
Unknown	1.436 (1.172‐1.760)	<0.001	0.966 (0.586‐1.593)	0.892	0.695 (0.357‐1.354)	0.285	0.468 (0.063‐3.492)	0.459
Chemotherapy								
No	Ref.	Ref.	Ref.		Ref.	Ref.	Ref.	
Yes	0.372 (0.304‐0.456)	<0.001	0.889 (0.707‐1.119)	0.316	1.682 (1.169‐2.420)	0.005	1.644 (1.021‐2.647)	0.041
Radiotherapy								
No	Ref.	Ref.	Ref.		Ref.	Ref.	Ref.	
Yes	0.367 (0.334‐0.402)	<0.001	0.586 (0.527‐0.651)	<0.001	0.448 (0.335‐0.600)	<0.001	0.563 (0.405‐0.783)	0.001

Abbreviations: BCT, Breast‐conserving therapy; ER, Estrogen receptor; PR, Progesterone receptor.

^a^ Multivariate analysis of surgery for OS was adjusted for age, race, marital status, T stage, N stage, ER, PR, chemotherapy, and radiotherapy.

^b^ Multivariate analysis of surgery for BCSS was adjusted for age, race, marital status, histologic grade, T stage, N stage, ER, PR, chemotherapy, and radiotherapy.

^†^ *P* < .05 was considered statistically significant.

### 3.3 Stratified analysis of overall survival

**FIGURE 2:**
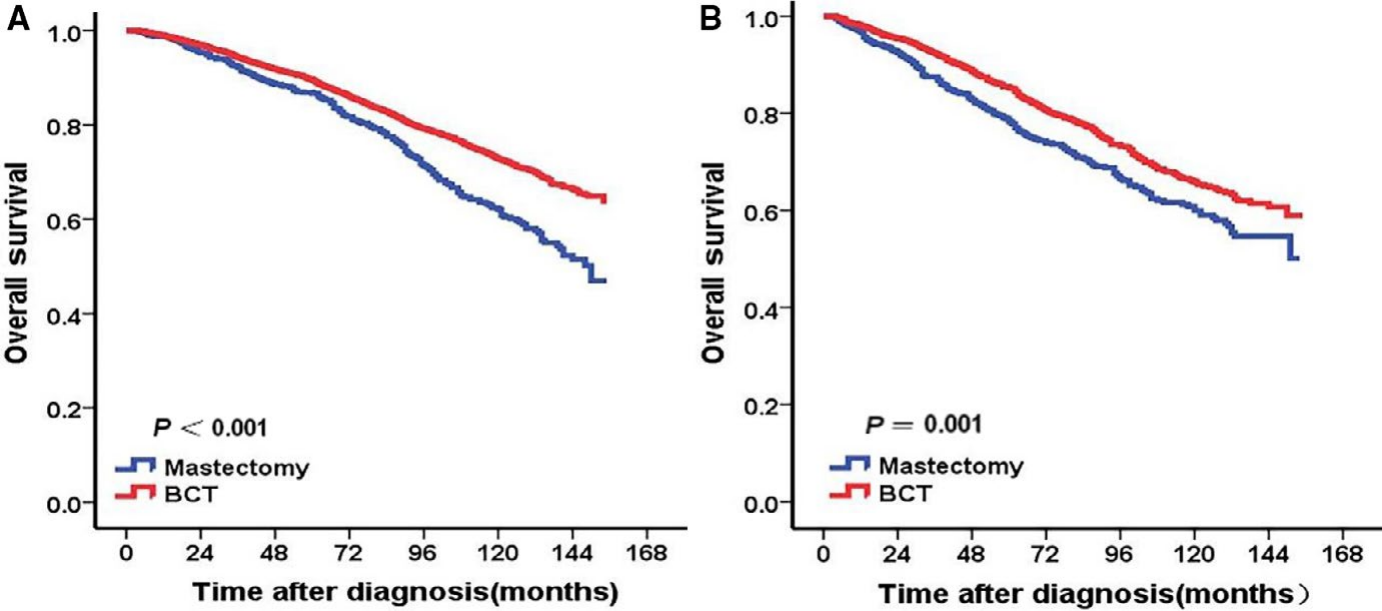
Kaplan‐Meier curves of overall survival for mucinous breast carcinoma patients according to surgical methods stratified by T stage. BCT group had better OS than mastectomy group for patients with T1 stage (A) or T2 stage (B)

**FIGURE 3:**
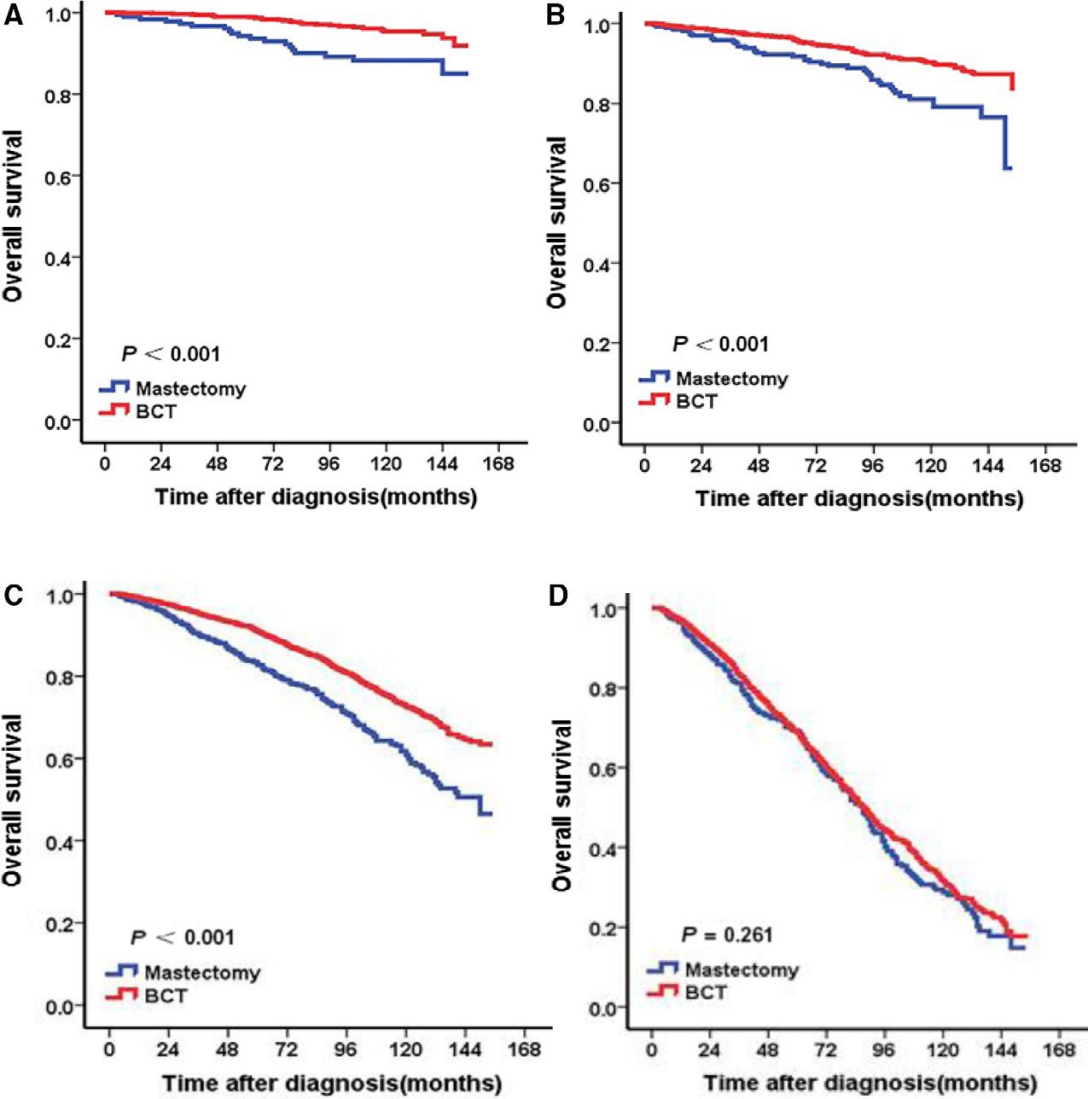
Kaplan‐Meier curves of overall survival for mucinous breast carcinoma patients according to surgical methods stratified by age. (A) Age of 23‐49 years. (B) Age of 50‐64 years. (C) Age of 65‐79 years. (D) Age ≥ 80 years. According to the different ages, BCT showed OS benefit in patients at the age of 50‐64 years (B) or age of 65‐79 years (C). There was no difference observed in OS between BCT and mastectomy groups for patients younger than 50 years (A) or not younger than 80 years (D)

**FIGURE 4:**
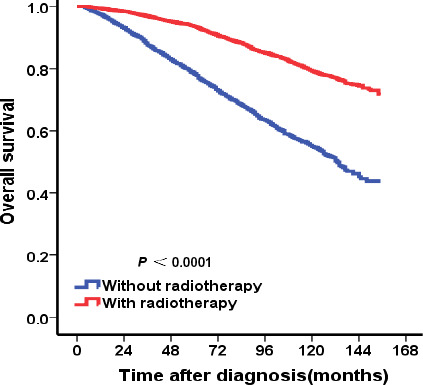
Kaplan‐Meier curves of overall survival for patients receiving BCT according to radiotherapy. For patients receiving BCT, the use of radiotherapy show better OS

As shown in Table 3, in stratified analysis according to T stage, adjusting for the significant prognostic variables in univariate analysis, multivariate analysis indicated that BCT group had better OS than mastectomy group for patients with T1 stage (HR = 0.679, 95% CI: 0.589‐0.781, *P* < .001)or T2 stage (HR = 0.769, 95% CI: 0.646‐0.915, *P* = .003) (Figure 2A and B). In stratified analysis according to the different ages, BCT showed OS benefit in patients at the age of 50‐64 years (HR = 0.587, 95% CI: 0.408‐0.846, *P* = .004) and age of 65‐79 years (HR = 0.636, 95% CI: 0.535‐0.758, *P* = .001) (Figure 3B and C). For patients not younger than 80 years, there was no difference significantly observed in OS between BCT and mastectomy groups (*P* > .05) (Figure 3D). For patients younger than 50 years, no difference significantly was observed in OS between BCT and mastectomy groups (HR = 0.576, 95% CI: 0.298‐1.115, *P* = .102) although Kaplan‐Meier curve showed patients in BCT group had better OS than those in mastectomy group (*P* < .05) (Figure 3A). For patients receiving BCT, the use of radiotherapy shows OS benefit (Figure 4).

**TABLE 3:** Stratified analysis of OS

Variable	OS			
	Univariate		Multivariate	
	HR（95%CI)	*P* ^†^	HR (95%CI)	*P* ^†^
Age (years)				
23‐49				
BCT vs Mastectomy	0.344 (0.192‐0.616)	<0.001	0.576 (0.298‐1.115)^a^	0.102
50‐64				
BCT vs Mastectomy	0.491 (0.348‐0.693)	<0.001	0.587 (0.408‐0.846)^b^	0.004
65‐79				
BCT vs Mastectomy	0.603 (0.510‐0.712)	<0.001	0.636 (0.535‐0.758)^c^	0.001
≥80				
BCT vs Mastectomy	0.913 (0.779‐1.070)	0.263	0.984 (0.836‐1.159)^d^	0.848
T stage				
T1				
BCT vs Mastectomy	0.667 (0.581‐0.766)	<0.001	0.679 (0.589‐0.781)^e^	<0.001
T2				
BCT vs Mastectomy	0.754 (0.633‐0.897)	0.001	0.769 (0.646‐0.915)^f^	0.003

Abbreviation: BCT, Breast‐conserving therapy.

^a^ HR for surgery was adjusted for the significant prognostic variables including marital status, T stage, N stage, chemotherapy, and radiotherapy.

^b^ HR for surgery was adjusted for the significant prognostic variables including marital status, T stage, ER, PR and, radiotherapy.

^c^ HR for surgery was adjusted for the significant prognostic variables including race, marital status, T stage, N stage, and radiotherapy.

^d^ HR for surgery was adjusted for the significant prognostic variables including marital status, T stage, ER, PR, and radiotherapy.

^e^ HR for surgery was adjusted for the significant prognostic variables including age, race, marital status, ER, PR, chemotherapy, and radiotherapy.

^f^ HR for surgery was adjusted for the significant prognostic variables including age, race, marital status, chemotherapy, and radiotherapy.

^†^ *P* < .05 was considered statistically significant.

## 4 DISCUSSION

Breast cancer is a histologically heterogeneous disease.^14^, ^15^, ^16^ Mucinous breast carcinoma is recognized as one of the most common rare histologic type in breast carcinoma. Experience in locoregional and systemic therapy of MBC was acquired from data of IDC and retrospective researches of MBC instead of prospective randomized control trials. There is limited evidence on the effect and safety of BCT in MBC compared with mastectomy. Therefore, we investigate the long‐term clinical outcomes of BCT and mastectomy in T1‐2 stage mucinous breast carcinoma using SEER database.

In our study, many more cases of MBC occurred in elderly and postmenopausal patients (88.2% older than 50 years). Furthermore, MBC cases had higher rates of ER or PR positivity (94% in ER positivity and 85% in PR positivity), lower histologic grade (56.2% in G1) and less lymph nodal metastases (93.5% without lymph nodal metastases). These observations are consistent with findings reported in previous western researches.^2^, ^3^, ^17^, ^18^, ^19^, ^20^

These features indicate that MBCs have a good prognosis. Therefore, some researchers have believed that BCT is an option for MBC which can minimize the trauma and maintain aesthetics. However, there is weak evidence concerning the safety and effect of treating MBC with less aggressive surgery such as BCT. A population‐based research from China was conducted to evaluate the impact of BCT in patients of pure MBC.^21^ In that study, 309 patients of pure mucinous breast carcinoma were reviewed from January 1, 1999 to October 1, 2010 and totally 64 patients underwent BCT (and radiotherapy). After a median follow‐up of 45.7 months, there was no statistically difference observed in the 5‐year OS rate between BCT group and mastectomy group (*P* = .096). Namely, OS was not influenced by the surgical methods.^21^ Another study analyzed 197 consecutive MBC patients, including 117 pure MBC and 80 mixed MBC, who were diagnosed between 1983 and 2014. No difference was observed in disease‐free survival in patients with pure MBC or patients with mixed MBC between two types of surgical treatment (HR = 0.077, 95% CI: 0.005‐1.227, *P* = .070; HR = 0.025, 95% CI: 0.000‐176.301, *P* = .414).^22^ In the current study, patients in BCT group had relatively better OS than those in mastectomy group. The risk of death from any cause in BCT group was lower than that in mastectomy group significantly (HR = 0.754, 95% CI: 0.697‐0.889, *P* < .001).While no difference significantly was observed in BCSS between BCT and mastectomy groups (HR = 1.232, 95% CI: 0.843‐1.800, *P* = .282).Our finding on OS is not in agreement with the results of above two studies. Our results showed it was safe and even better to receive BCT for MBC patients. The controversy among these studies might be owing to the short follow‐up time and small sample sizes of previous studies. Besides, our study focused on the patients with T1‐2 stage (tumor size ≤ 5 cm), which meant tumor size could meet the BCT indication and most of these patients had opportunities to choose BCT. We also found that in stratified analysis according to T stage, BCT group had better OS than mastectomy group for patients with either T1 stage or T2 stage. As shown in Table 2, we first conducted univariate analysis and the results demonstrated that BCT group had both better OS (*P* < .001) and BCSS (*P* = .004). However, the results were not consistent in multivariate analyses. We think the bias might have two origins. First, the tracking system of SEER database could make mistakes in recording some cancer‐specific deaths. Second and more importantly, the percentages of other treatments were not equal in the two groups. BCT group had a higher rate of radiotherapy while mastectomy group accepted more chemotherapy. Given the most eligible patients in the present study were elder females at a median age of 67 years, the therapy‐associated comorbidities may bring great bias in the evaluation of BCSS. To clarify the effect of BCT on BCSS, further prospective clinical trials might be needed.

Radiotherapy, as an important adjuvant therapy, is widely used in invasive breast cancer patients after breast‐conserving therapy or in patients with high‐risk factors after mastectomy.^11^ Therefore, radiotherapy may be important in MBC. However, the clinical value of radiotherapy in MBC remains unclear. In our study, in BCT group a total of 5038 (67.1%) patients received radiotherapy and 2472 (32.9%) did not receive radiotherapy. The use of radiotherapy shows OS benefit for them.

MBC is classified into two main subtypes histologically: pure mucinous breast cancer (PMBC) when the mucinous component accounts for more than 90% and mixed mucinous breast cancer (MMBC) when there is 10%‐49% non‐mucinous co‐existing cancer component in the tumor. MMBC can be classified into different subtyped according to co‐existing cancer component.^22^, ^23^, ^24^, ^25^, ^26^ Therefore the prognosis of different subtypes may be different. The cases of above studies ^21^, ^22^ concerning the prognosis of BCT and mastectomy were PMBC or MMBC. In present study, we did not analyze the data stratified by different subtypes. Further analysis stratified according to histologic subtypes is needed to determine the exact prognosis of BCT and mastectomy.

Our study still had several limitations. Firstly, other known prognostic information such as lymphovascular invasion was not available. Secondly, the bias of surgical method selection was inevitable, because the selection was determined not only by tumor size but also by the preferences of patient or surgeon. Thirdly, the data on endocrine therapy could not be available in SEER database. Although the percentages of receiving standard endocrine therapy between the two groups is unknown, we could estimate the percentages of endocrine therapy depending on the rates of ER/PR + in the two groups, since most MBC patients were hormone receptor positive and they who completed appropriate locoregional treatment were likely to receive standard endocrine therapy. As shown in Table 1, BCT group had higher ER/PR + rates than mastectomy group (*P* < .001), which implied there were more patients accepting endocrine therapy in BCT group. To decrease the bias, we have employed the ER/PR in the final multivariate analyses of prognostic factors. The results demonstrated that BCT still occupied a better overall survival. So we think the results are reliable.

Therefore, further prospective clinical trials are warranted to evaluate the exact prognosis and safety of BCT and mastectomy in MBC.

## 5 CONCLUSIONS

This large population‐based study indicated patients who received BCT had better prognosis than those received mastectomy in T1‐2 stage MBC, especially in patients at the age of 50‐79 years. The use of radiotherapy showed OS benefit in patients receiving BCT. Breast‐conserving therapy might be preferred over mastectomy especially in locoregional treatment of T1‐2 stage MBC.

## CONFLICT OF INTEREST

The authors declare to have no competing interest.

## AUTHORS’ CONTRIBUTIONS

Ping Yu, Feng Ye and Xiaoming Xie conceived the research and wrote the manuscript. Peng Liu and Yutian Zoutook charge of data interpretation and language editing. Yutian Zou and Na Li participated in collection of data. Hailing Tang and Xinhua Xie performed all data analysis. All the authors were involved in approval of the final version.

## CONSENT FOR PUBLICATION

Not applicable.

## ETHICS APPROVAL AND CONSENT TO PARTICIPATE

All procedures performed in studies involving human participants were in accordance with the ethical standards of the institutional and/or national research committee and with the 1964 Helsinki declaration and its later amendments or comparable ethical standards. This study was approved by the Research Ethics Committee of Sun Yat‐sen University Cancer Center.

## Supporting information

Figure S1

## Data Availability

All the data were extracted from the Surveillance, Epidemiology and End Results (SEER) database released in November 2018. Qualified researchers may access to information on cancer statistics through the website of SEER database (https://seer.cancer.gov/).

## References

[1] Bae SY , Choi MY , Cho DH , Lee JE , Nam SJ , Yang JH . Mucinous carcinoma of the breast in comparison with invasive ductal carcinoma: clinicopathologic characteristics and prognosis. J Breast Cancer. 2011;14:308‐313. 22323918 10.4048/jbc.2011.14.4.308 PMC3268928

[2] Di Saverio S , Gutierrez J , Avisar E . A retrospective review with long term follow up of 11,400 cases of pure mucinous breast carcinoma. Breast Cancer Res Treat. 2008;111:541‐547. 18026874 10.1007/s10549-007-9809-z

[3] Hanagiri T , Ono K , Baba T , et al. Clinicopathologic characteristics of mucinous carcinoma of the breast. Int Surg. 2010;95:126‐129. 20718318

[4] Li CI , Uribe DJ , Daling JR . Clinical characteristics of different histologic types of breast cancer. Br J Cancer. 2005;93:1046‐1052. 16175185 10.1038/sj.bjc.6602787 PMC2361680

[5] Zhang M , Teng XD , Guo XX , Zhao JS , Li ZG . Clinicopathological characteristics and prognosis of mucinous breast carcinoma. J Cancer Res Clin Oncol. 2014;140:265‐269. 24305754 10.1007/s00432-013-1559-1 PMC11823925

[6] Ohashi R , Sakatani T , Matsubara M , et al. Mucinous carcinoma of the breast: a comparative study on cytohistological findings associated with neuroendocrine differentiation. Cytopathology. 2016;27:193‐200. 26804749 10.1111/cyt.12298

[7] Diab SG , Clark GM , Osborne CK , Libby A , Allred DC , Elledge RM . Tumor characteristics and clinical outcome of tubular and mucinous breast carcinomas. J Clin Oncol. 1999;17:1442‐1448. 10334529 10.1200/JCO.1999.17.5.1442

[8] Vo T , Xing Y , Meric‐Bernstam F , et al. Long‐term outcomes in patients with mucinous, medullary, tubular, and invasive ductal carcinomas after lumpectomy. Am J Surg. 2007;194:527‐531. 17826073 10.1016/j.amjsurg.2007.06.012

[9] Tseng HS , Lin C , Chan SE , et al. Pure mucinous carcinoma of the breast: clinicopathologic characteristics and long‐term outcome among Taiwanese women. World J Surg Oncol. 2013;11:139. 23768133 10.1186/1477-7819-11-139 PMC3689603

[10] Clarke M , Collins R , Darby S , et al. Effects of radiotherapy and of differences in the extent of surgery for early breast cancer on local recurrence and 15‐year survival: an overview of the randomised trials. Lancet. 2005;366:2087‐2106. 16360786 10.1016/S0140-6736(05)67887-7

[11] Fisher B , Anderson S , Bryant J , et al. Twenty‐year follow‐up of a randomized trial comparing total mastectomy, lumpectomy, and lumpectomy plus irradiation for the treatment of invasive breast cancer. N Engl J Med. 2002;347:1233‐1241. 12393820 10.1056/NEJMoa022152

[12] Barkley CR , Ligibel JA , Wong JS , Lipsitz S , Smith BL , Golshan M . Mucinous breast carcinoma: a large contemporary series. Am J Surg. 2008;196:549‐551. 18809061 10.1016/j.amjsurg.2008.06.013

[13] Thurman SA , Schnitt SJ , Connolly JL , et al. Outcome after breast‐conserving therapy for patients with stage I or II mucinous, medullary, or tubular breast carcinoma. Int J Radiat Oncol Biol Phys. 2004;59:152‐159. 15093911 10.1016/j.ijrobp.2003.10.029

[14] Cancer Genome Atlas N . Comprehensive molecular portraits of human breast tumours. Nature. 2012;490:61‐70. 23000897 10.1038/nature11412 PMC3465532

[15] Perou CM , Sorlie T , Eisen MB , et al. Molecular portraits of human breast tumours. Nature. 2000;406:747‐752. 10963602 10.1038/35021093

[16] Akiyama F , Horii R . Therapeutic strategies for breast cancer based on histological type. Breast Cancer. 2009;16:168‐172. 19479319 10.1007/s12282-009-0126-8

[17] Avisar E , Khan MA , Axelrod D , Oza K . Pure mucinous carcinoma of the breast: a clinicopathologic correlation study. Ann Surg Oncol. 1998;5:447‐451. 9718175 10.1007/BF02303864

[18] Fentiman IS , Millis RR , Smith P , Ellul JP , Lampejo O . Mucoid breast carcinomas: histology and prognosis. Br J Cancer. 1997;75:1061‐1065. 9083343 10.1038/bjc.1997.180 PMC2222746

[19] Skotnicki P , Sas‐Korczynska B , Strzepek L , et al. Pure and mixed mucinous carcinoma of the breast: a comparison of clinical outcomes and treatment results. Breast J. 2016;22:529‐534. 27261206 10.1111/tbj.12621

[20] Louwman MW , Vriezen M , van Beek MW , et al. Uncommon breast tumors in perspective: incidence, treatment and survival in the Netherlands. Int J Cancer. 2007;121:127‐135. 17330844 10.1002/ijc.22625

[21] Cao AY , He M , Liu ZB , et al. Outcome of pure mucinous breast carcinoma compared to infiltrating ductal carcinoma: a population‐based study from China. Ann Surg Oncol. 2012;19:3019‐3027. 22451233 10.1245/s10434-012-2322-6

[22] Pan B , Yao R , Shi J , et al. Prognosis of subtypes of the mucinous breast carcinoma in Chinese women: a population‐based study of 32‐year experience (1983–2014). Oncotarget. 2016;7:38864‐38875. 27102151 10.18632/oncotarget.8778 PMC5122436

[23] Laucirica R , Bentz JS , Khalbuss WE , Clayton AC , Souers RJ , Moriarty AT . Performance characteristics of mucinous (colloid) carcinoma of the breast in fine‐needle aspirates: observations from the College of American Pathologists Interlaboratory Comparison Program in Nongynecologic Cytopathology. Arch Pathol Lab Med. 2011;135:1533‐1538. 22129179 10.5858/arpa.2010-0652-CP

[24] Silverberg SG , Kay S , Chitale AR , Levitt SH . Colloid carcinoma of the breast. Am J Clin Pathol. 1971;55:355‐363. 4323690 10.1093/ajcp/55.3.355

[25] Ding S , Wu J , Lin C , et al. Predictors for survival and distribution of 21‐gene recurrence score in patients with pure mucinous breast cancer: a SEER population‐based retrospective analysis. Clin Breast Cancer. 2019;19:e66‐e73. 30396812 10.1016/j.clbc.2018.10.001

[26] Komaki K , Sakamoto G , Sugano H , Morimoto T , Monden Y . Mucinous carcinoma of the breast in Japan. A prognostic analysis based on morphologic features. Cancer. 1988;61:989‐996. 2827884 10.1002/1097-0142(19880301)61:5<989::aid-cncr2820610522>3.0.co;2-e

