# Supplementary figures and images for: Breast‐conserving therapy shows better prognosis in mucinous breast carcinoma compared with mastectomy: A SEER population‐based study
Source: Cancer Med. 2020 Jun 8;9(15):5381–91. doi: 10.1002/cam4.3202 (PMC7402828; doi:10.1002/cam4.3202)

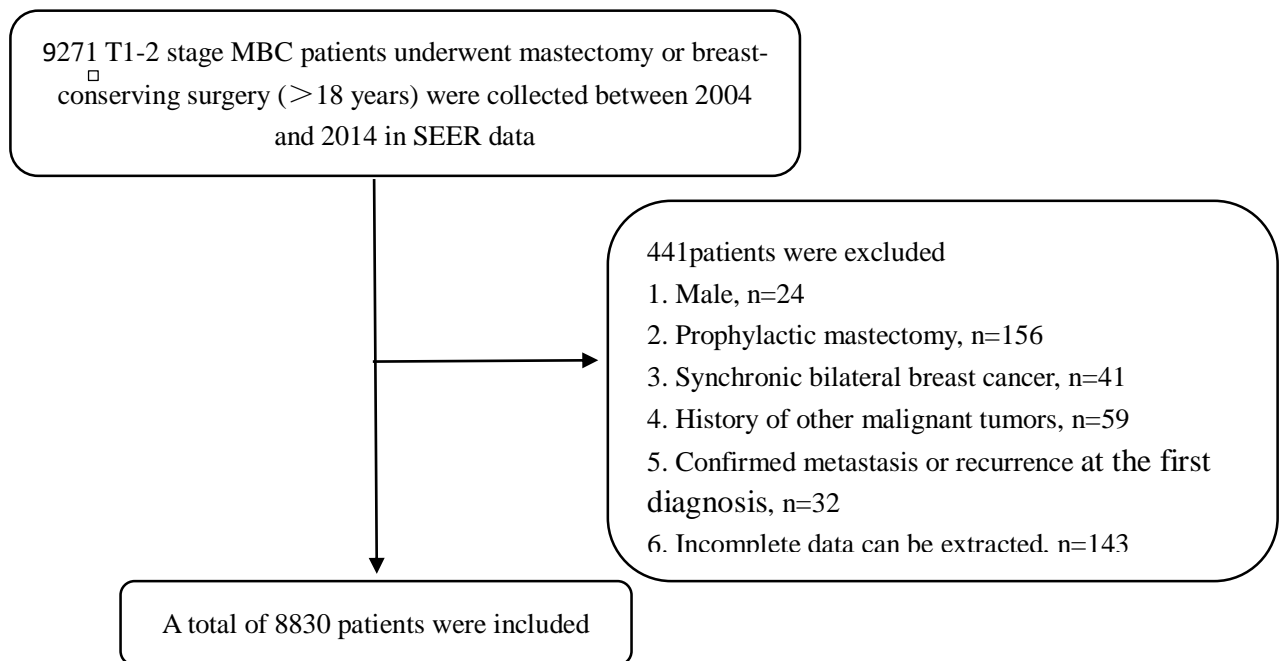

Supplementary Fig. 1. Flow diagram of patient selection.

Supplement: Supplementary file 1 — Figure S1 [file CAM4-9-5381-s001.pdf]
